# Metabolic Shift Mirrors GBM Immunity to Anti-PD-L1 Immunotherapy: A Deuterium MRS Study

**DOI:** 10.1007/s11307-025-02037-w

**Published:** 2025-10-03

**Authors:** Joel R. Garbow, Xia Ge, Tanner M. Johanns, John A. Engelbach, Keith M. Rich, Joseph J. H. Ackerman

**Affiliations:** 1 Department of Radiology, Washington University School of Medicine, St. Louis, MO 63110 USA; 2 Department of Medicine, Washington University School of Medicine, St. Louis, MO 63110 USA; 3 Alvin J Siteman Cancer Center, Washington University School of Medicine, St. Louis, MO 63110 USA; 4 Department of Neurosurgery, Washington University School of Medicine, St. Louis, MO 63110 USA; 5 Department of Chemistry, Washington University School of Medicine, St. Louis, MO 63130 USA

**Keywords:** MRI, MRS, Radiation, Tumor, Microenvironment, Immunotherapy, Checkpoint inhibitors, Deuterium

## Abstract

**Background/Objectives:**

Immune checkpoint blockade (ICB) therapy has been ineffective in glioblastoma (GBM) that recurs following standard-of-care resection and chemoradiation of the primary tumor. Herein, we investigate whether the delayed effect of intracranial radiation alters the tumor lesion metabolic profile.

**Methods:**

Naïve (non-irradiated) GL261 tumor cells were implanted into the brains of C57BL/6 mice. Brains of one cohort were hemispherically irradiated six weeks prior to implantation, ultimately resulting in ICB refractory GBM. Brains of the control cohort were not irradiated. Following subcutaneous infusion of [6,6-^2^H_2_] glucose (Glc), single voxel deuterium metabolic imaging (DMI) monitored Glc uptake and the production of semi-heavy water (HOD), ^2^H_2_-lactate (Lac) and the 50/50 mix of [^2^H_2_-glutamate + ^2^H_2_-glutamine] (Glx).

**Results:**

GL261 tumors growing in previously irradiated brain showed reduced Warburg effect (aerobic glycolysis; glucose → lactate) and greater TCA cycle activity (respiration, oxidative phosphorylation) relative to tumors growing in non-irradiated brain as evidenced by cohort differences in the ratios Glx/Lac (p < 0.01), Glx/Glc (p < 0.02), and Lac/Glc (p < 0.01).

**Conclusions:**

A metabolic program skewed toward oxidative phosphorylation and away from glycolysis has been associated with immune dysfunction. This study documents such a skewed metabolic state in ICB refractory GL261 GBM growing in irradiated brain (tumors were not irradiated) compared to control brain.

## Introduction

Chemoradiation is the standard-of-care therapy for newly diagnosed glioblastoma (GBM). However, GBM invariably recurs, with no effective treatment options. Immune checkpoint blockade (ICB) therapy has been ineffective in GBM in recurrent settings, but the basis for resistance is unclear. Recently, we observed that implantation of naïve (non-irradiated) GL261 cells, a highly ICB-responsive murine GBM cell line, into a brain microenvironment irradiated six weeks previously results in complete resistance to ICB therapy [1]. Herein, in the same animal model, we investigate whether the delayed effect of intracranial radiation alters the tumor lesion metabolic profile, as monitored by deuterium MR imaging/spectroscopy (DMI).

There have been several clinical reports suggesting ICB treatment of brain metastases is less effective in lesions that were previously treated with intracranial radiation [2–4]. Prolonged standard-of-care GBM irradiation may likewise contribute to the negative results for ICB therapy in GBM [5–7]. Similarly, while ICB therapy has demonstrated activity in first-line treatment of metastatic squamous cell carcinoma of the head and neck (HNSCC), in which radiation is not used [8], the addition of ICB therapy to a prolonged seven-week course of HNSCC chemoradiation confers no benefit over chemoradiation alone [9]. Together, these data suggest prolonged radiation or delayed effects of radiation may mitigate the potential clinical benefits of ICB treatment in brain and other solid tumors.

Intrigued by this hypothesis, we investigated whether DMI quantification of metabolic activity would distinguish an ICB-nonresponsive GBM mouse cohort from an ICB-responsive cohort where the only difference between cohorts was ± brain pre-irradiation six weeks prior to implantation of naïve (non-irradiated) GL261 cells. We have used DMI in irradiated mouse brain/tumor to distinguish tumor from radiation necrosis (RN) [10] and to quantify %tumor in a mixed tumor/RN lesion [11, 12]. DMI [10–18] monitors the metabolic products of deuterated substrates, and of specific relevance herein, the intracellular conversion of deuterated glucose ([6,6-^2^H_2_]glucose; Glc) to [3,3-^2^H_2_]lactate (Lac) and the sum of a nominal 50/50 mix of [4,4-^2^H_2_]glutamine + [4,4-^2^H_2_]glutamate (Glx). DMI determined concentrations of Glx and Lac provide an assessment of the relative contributions of oxidative phosphorylation (TCA cycle) vs aerobic glycolysis (AG; Warburg effect, glucose → lactate) pathways, e.g., ratio of [Lac]/[Glx].

## Materials and Methods

### Gamma Knife Animal Irradiation

Mice were irradiated using the Leksell Gamma Knife® (GK) Icon™ unit (Elekta, Inc. Stockholm, Sweden), a gamma-ray radiosurgery device. The brains of irradiated mice received focal hemispheric GK irradiation (30 Gy × 1, 50% isodose) 6 weeks prior to GL261 cell implantation. Mice whose brains are irradiated with 30 Gy show no signs of behavioral, anatomic (MRI), or histologic (H&E) change vs control (non-irradiated) for 10 weeks post irradiation [1]. In unpublished findings, we have observed such a lack of effect extending to 6 months post irradiation.

### GL261 Tumors and Tumor-Cell Implantation

GL261 GBM cells (~ 50,000 cells suspended in 10 μL per mouse) were orthotopically implanted according to standard procedures [19]. Tumor growth was monitored by ^1^H MRI, vide infra, and DMI measurements were generally made 2–4 weeks post implantation of GL261 cells, with tumor volumes typically in the range of 60–90 μL.

### MR Characterization and Immune Profiling

A previous report from this lab described multi-contrast MRI assessment of GL261 tumor implanted in brain-irradiated and non-irradiated C57/BL6 mice, providing detailed anatomical and functional imaging characterization [11]. MRI contrasts included T1-weighted (T1W), T2-weighted (T2W), and contrast enhanced (CE) images, and maps of R1, R2, apparent diffusion coefficient (ADC) and magnetization transfer ratio (MTR). We have also reported histology, immunohistochemistry, and FACS analysis of this irradiated brain model [1], Differences in the relative frequency of myeloid subpopulations were observed in GL261 brain tumors growing in previously-brain-irradiated vs non-brain-irradiated mice. Specifically, there was a significant difference in the relative frequency of activated vs resting microglia in the previously-irradiated, tumor-bearing mice.

### Magnetic Resonance Imaging

For all ^1^H and ^2^H imaging experiments: (i) mice were anesthetized with isoflurane and placed in a stereotactic head holder to minimize motion, and (ii) contrast agent (100 μL of a 50% saline dilution of Dotarem) was administered out-of-magnet intraperitoneally. *Anatomic *^*1*^*H MRI:* Mice were imaged longitudinally on 4.7-T (200-MHz) or 9.4-T (400 MHz) small-animal scanners post-implantation of tumors. Standard contrast-enhanced (CE) transaxial T1W and T2W images were collected for quantitative monitoring of lesion volumes. *T1W images:* T1W images were acquired with a 2D spin-echo multi-slice (SEMS) sequence: TR = 0.6 s, TE = 11 ms, FOV = 16 × 16 mm^2^, matrix size = 128 × 128, 4 averages. *T2W images:* T2W images were acquired with a 2D fast spin-echo multi-slice (FSEMS) sequence: TR = 2 s, echo train length (ETL) = 4, Kzero = 4, effective TE = 52 ms; FOV = 16 × 16 mm^2^, matrix size = 128 × 128, 6 averages. *Deuterated Substrate (Glucose) Administration:* [6,6-^2^H_2_]glucose (Glc) was administered in-magnet subcutaneously (sc) [10] at a dose of 4.5 g/kg body weight. *Deuterium Metabolic Imaging:* Single voxel DMI data were acquired on a small-animal 11.74-T MRI scanner with the *Sp*in-*Ec*ho Full-*I*ntensity *A*cquired *L*ocalized Spectroscopy (SPECIAL) pulse sequence [20] in concert with outer volume suppression [21]. Coronal and transaxial FSEMS ^1^H MR images, TR = 600 ms, TE = 8.8 ms, identified the location of tumors for placement of the DMI voxel. DMI free induction decays (FIDs) were acquired in 8 consecutive 10-min signal-averaging blocks. The initial 10-min acquisition block occurred immediately prior to subcutaneous administration of Glc and served to reference the natural abundance semi-heavy water (^1^HO^2^H = HOD) signal amplitude (= 16.35 mM [11, 22]), a convenient internal standard. The repeated 10-min acquisitions quantified the conversion of Glc to Lac and Glx.

### Data Analysis

The deuterium resonance amplitudes for Glc, Glx, Lac and HOD were determined using Bayesian-based analysis software [23]. The amplitudes for Glc, Glx, and Lac, proportional to species concentrations, were adjusted for relaxation effects and stoichiometry/label loss [24]. The Glx/Lac metabolite concentration ratio is often used as a measure of TCA cycle vs AG metabolism. In literature focused on blood-flow perfusion measurements, labeled water (e.g., H_2_^15^O) is recognized as a flow-limited diffusible tracer [25–27]. Thus, in DMI studies, HOD produced intracellularly from downstream metabolism of administered substrate will distribute throughout the body once the HOD exits the intracellular milieu and enters the vascular system. For this reason, it is likely that the HOD content observed in brain has contributions from HOD produced by substrate metabolism elsewhere in the body.

Statistical differences between cohorts (GL261 tumors growing in brain-irradiated vs non-brain irradiated mice) were accepted at the 95% confidence limit, p < 0.05 via standard t-test.

## Results

**Fig. 1:**
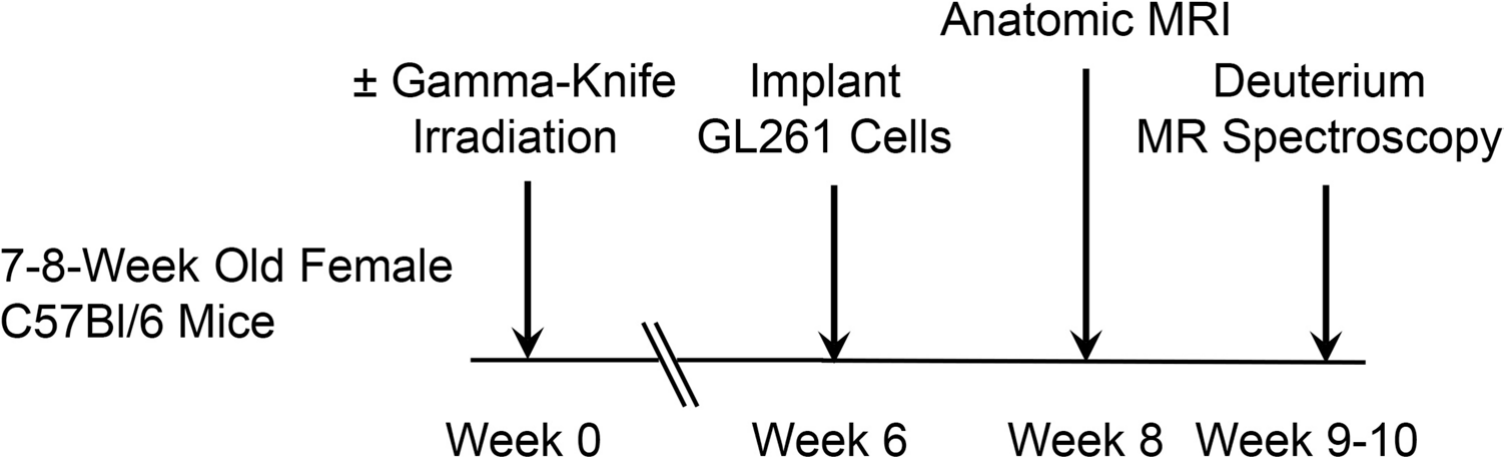
Experimental timeline

**Fig. 2:**
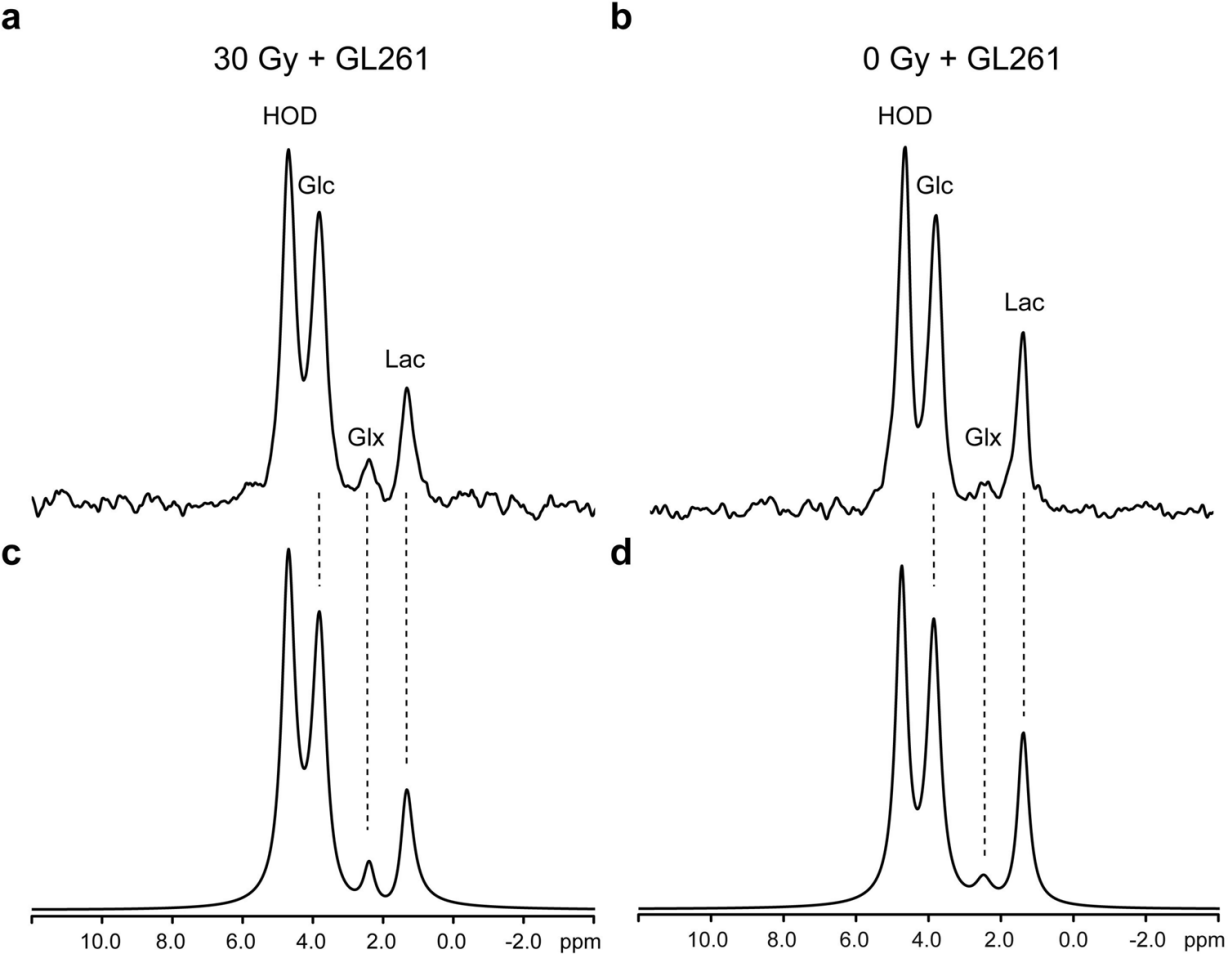
**(a, b)** Exemplary mouse brain-tumor DMI spectra representing the sum of seven 10-min SPECIAL acquisitions (70 min total) initiated immediately post sc administration of Glc (4.5 g/kg). **(c, d)** Results of Bayesian spectral modeling are shown below the spectra. Note the elevated Glx and reduced Lac in the GL261 tumor implanted in pre-irradiated brain **(a, c)**

**Fig. 3:**
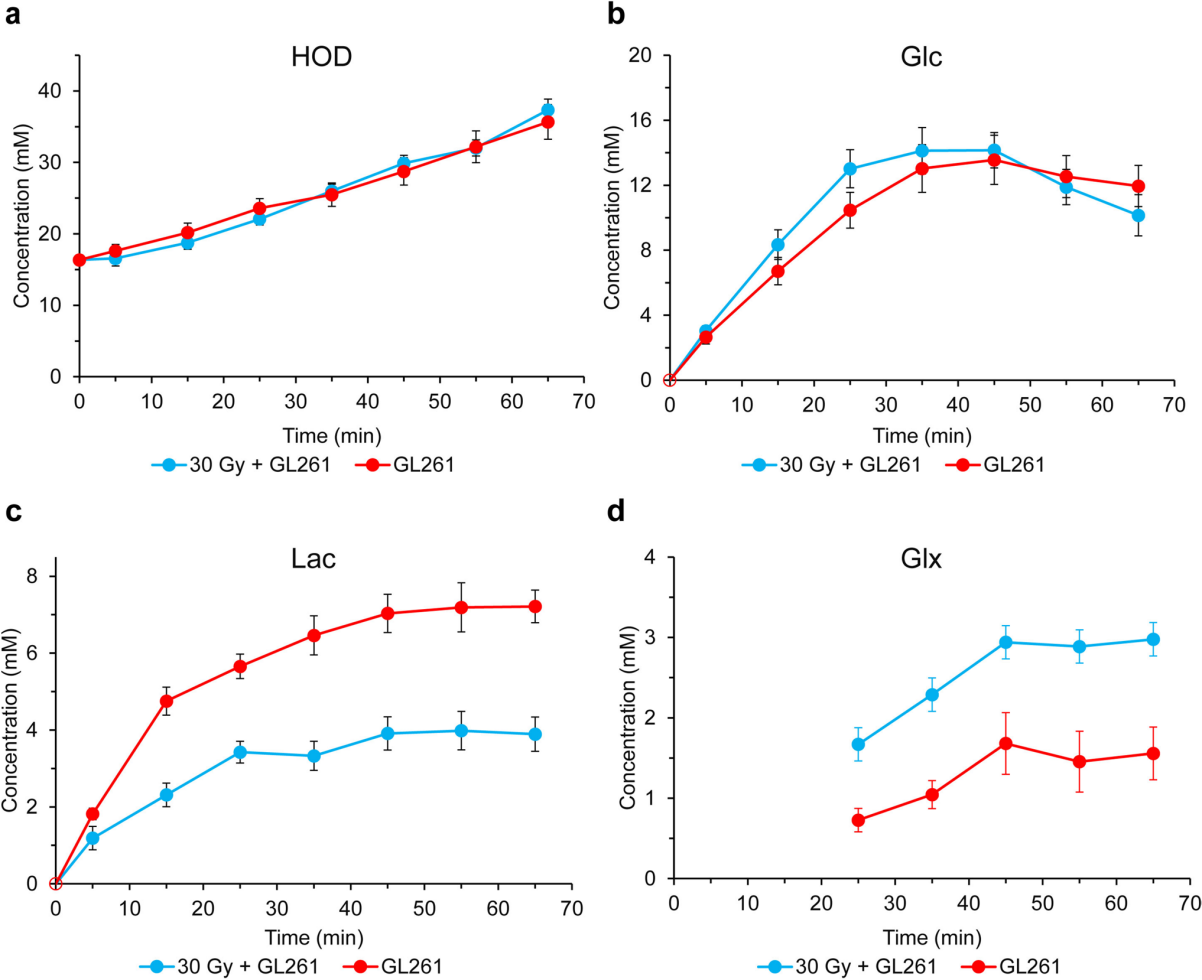
Time-course plots of **(a)** HOD, **(b)** Glc, **(c)** Glx, and **(d)** Lac following sc administration of Glc (4.5 g/kg). for GL261 tumor implanted in non-irradiated brain (n = 13, red) and GL261 tumor in brain irradiated (30 Gy) 6 weeks prior to tumor implantation (n = 7, blue). Seven 10-min SPECIAL acquisitions were initiated immediately post sc administration of Glc. Data points represent the mean over all animals in a cohort; error bars are standard errors of the mean. The Glx signal from the 10-min data acquisitions collected prior to 20-min post-injection are at-or-below the noise floor and, therefore, are not displayed

Figure 1 shows the experimental timeline for these studies, including mouse brain irradiation, tumor implantation, anatomic MR imaging and deuterium MR spectroscopy. Figure 2 displays spectra acquired from individual members of the two cohorts of mice bearing GL261 brain tumors. Spectra are the sums of seven 10-min acquisitions (70 min total; time-courses shown in Fig. 3). The upper left panel (a) shows the DMI spectrum from a GL261 tumor implanted in pre-irradiated brain; the upper right panel (b) shows the DMI spectrum from a GL261 tumor implanted in non-irradiated brain. Results of Bayesian spectral modeling are shown below the spectra (c, d). The amplitude of the Glx signal is clearly elevated, and Lac reduced, in the tumor implanted in pre-irradiated brain (a) vs tumor implanted in non-irradiated brain (b). Note that the observed HOD signal amplitude is far greater than that of natural-abundance HOD, as water is also a metabolic end-product.

Figure 3 shows cohort-averaged, time-course, dynamic (kinetic) plots of the uptake and metabolism of glucose for irradiated (blue) and non-irradiated (red) tumor following sc administration of Glc (4.5 g/kg). Seven 10-min SPECIAL datasets were acquired immediately post sc administration of Glc. Data points represent the mean over all animals in a cohort.

**Fig. 4:**
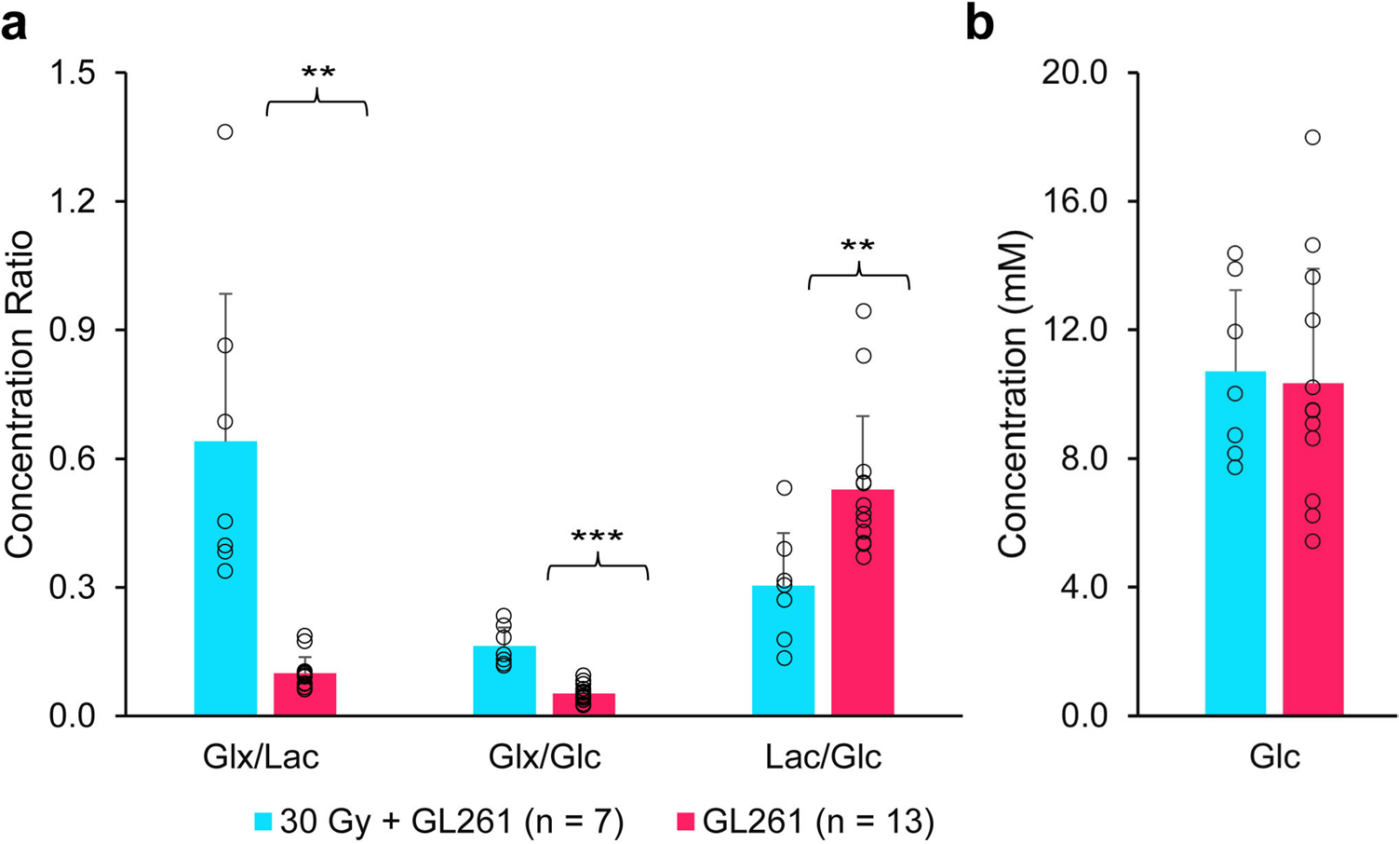
A bar graph summary (mean and standard error of the mean) of **(b)** glucose substrate concentration (Glc) and **(a)** ratios (Glx/Lac, Glx/Glc, and Lac/Glc; for GL261 tumor implanted in non-irradiated brain (n = 13, red) and GL261 tumor in brain irradiated (30 Gy) 6 weeks prior to tumor implantation (n = 7, blue). Individual data points are superimposed on each bar graph. T test: ** *p* < 0.01, *** *p* < 0.002

Figure 4 displays a bar graph summary (mean, standard deviation) of Glc concentration (far right panel, b) and concentration ratios (Glx/Lac, Glx/Glc, and Lac/Glc; left panels, a) for GL261 tumor implanted in non-irradiated brain (n = 13, red) and GL261 tumor in brain irradiated (30 Gy) 6 weeks prior to tumor implantation (n = 7, blue). Mean Glc concentrations were essentially equivalent in the two cohorts. As anticipated, substantial conversion of Glc to Lac (AG) is observed in both tumor cohorts. However, the Glx/Lac metabolite concentration ratio, an assay of TCA cycle vs AG metabolism, is significantly greater in tumor implanted in pre-irradiated brain.

We note that intersubject variability in the deuterium MR metabolic assays can be ascribed to several factors. (i) Mice were fed ad libitum (i.e., were not fasted) and were scanned across all hours of the normal working day, i.e., 8 am – 5 pm. Thus, a specific mouse may or may not have eaten shortly prior to scanning and cohort members reflect variable fed vs fasted states. (ii) GL261 tumors, like most cancers, can present a heterogeneous tissue microenvironment with regions of normoxia, hypoxia, and necrosis. Thus, cohort members present a range of tumor physiologic status. (iii) Alignment of the tumor implantation site with the focal position of the Gamma Knife irradiation is imperfect. Thus, GL261 tumors growing in cohort members result from cells implanted in non-identically irradiated brain microenvironments.

## Discussion

Aerobic glycolysis is a signature metabolic phenotype of malignant tumors, characterized by high glycolytic activity, resulting in lactate production (fermentation) even in the presence of oxygen [28, 29]. This contrasts with normal/differentiated cells, in which glucose is preferentially converted to lactate only under hypoxic conditions, a process referred to as anaerobic glycolysis [30, 31].

It has been postulated that AG provides key components for cellular growth, including ATP for energy, intermediate metabolites necessary for lipid synthesis, ribose for nucleoside synthesis, and enhanced levels of glutathione, thereby augmenting reducing equivalents to counteract oxidative stress [30]. Tumor AG-derived lactate and protons actively secreted extracellularly by monocarboxylate transporters are key components enabling production of the acidic tumor microenvironment and promoting increased tumor aggressiveness [32–34].

Until recently, a focus on AG has led to downplaying the role of other metabolic pathways, especially the TCA cycle, in cancer. However, modulated/abnormal TCA cycle metabolism occurs in many pathological processes and there is growing realization that a variety of cancers also employ the TCA cycle for energy production and macromolecule biosynthesis [35]. Indeed, evidence is accumulating in support of the important role of the TCA cycle in cancer metabolism and tumorigenesis [36].

Changes in the metabolic state of immune cells can influence their effector function and role in anti-cancer immunity. For example, stimulation of macrophages in vitro with LPS ± IFNg to derive an inflammatory, anti-tumor M1 phenotype results in induction of glycolysis, whereas stimulation with IL-4 to generate an immunosuppressive, pro-tumorigenic M2 phenotype induces an increase in oxidative phosphorylation [37]. A similar association of preferential glycolysis with a pro-inflammatory state and oxidative phosphorylation with an anti-inflammatory state has been described for microglia [38]. In addition to myeloid cells, T-cell effector function has also been attributed to differential metabolic states [39]. For instance, activated T cells are characterized by a highly glycolytic metabolic state, whereas T cells in a quiescent state and exhausted T cells are associated preferentially with oxidative phosphorylation [39]. Interestingly, regulatory T cells and monocyte-derived suppressor cells also preferentially use oxidative phosphorylation [39] for energy metabolism. These data demonstrate that altered metabolic programming is associated with differential effector states of immune cells, with a preferential utilization of oxidative phosphorylation over glycolysis in a dysfunctional immune state.

In our mouse brain tumor model, the brain microenvironment is modulated via focal irradiation (30 Gy) from a GK unit and presents a GBM phenotype that is refractory to ICB therapy. Use of this model offered a novel test case for DMI assessment of a putative glycolysis/TCA metabolic shift as regards the immune response in cancer therapy.

Our data report the remarkable finding that the two GL261 GBM models, differing only in a radiation-modulated brain microenvironment prior to cell implantation, show not only strikingly different ICB responses [1], but also accompanying differences in DMI-assayed TCA cycle vs AG metabolism. The pre-irradiated GL261 GBM model is ICB refractory [1] and has increased TCA cycle and decreased AG metabolism. We hypothesize that a DMI assessment of a shift away from AG is indicative of a tumor that will not respond to ICB.

## Conclusions

Several mechanisms have been proposed to explain the lack of clinical efficacy observed with ICB in patients with recurrent GBM, including: i) predominance of immunosuppressive cells or secretion of immunosuppressive cytokines, ii) lack of or deficient effector cell populations, iii) tumor cell heterogeneity, and iv) various strategies of immune evasion intrinsic to tumor cells [40]. We have developed a mouse model [1] in which previous (6 weeks) focal brain irradiation results in aggressive growth and non-responsiveness to anti-PD-L1 immunotherapy, mimicking recurrent GBM in the clinic. Deuterium MRI data show these effects are mirrored by increased TCA cycle activity and reduced aerobic glycolysis, consistent with reports by others that a dysfunctional immune state is characterized by preferential utilization of oxidative phosphorylation over glycolysis.

## Data Availability

All research data and computer codes are available from the corresponding author upon request.
